# Dataset and detailed methodology for structure and performance characterization of modified polymeric membranes

**DOI:** 10.1016/j.dib.2019.104862

**Published:** 2019-11-22

**Authors:** Raphael Rodrigues, José Carlos Mierzwa, Chad D. Vecitis

**Affiliations:** a Department of Hydraulic and Environmental Engineering, Polytechnic School, University of Sao Paulo, Sao Paulo, SP, 05508-900, Brazil; b Harvard John A. Paulson School of Engineering and Applied Sciences, Harvard University, Cambridge, MA, 02138, United States; c Department of Civil and Environmental Engineering, Rensselaer Polytechnic Institute, 110 8th Street, Troy, NY, 12180, United States

**Keywords:** Membrane characterization, Membrane performance, Membrane properties

## Abstract

The data contained in this publication refers to protocols adopted characterization of clay nanoparticles (CN) membranes with and without the use of polyethylene oxide (PEO) as pore former. The membrane casting solutions were produced by dissolving PS (18% w/w) in NMP with addition of CN (1–5% w/w CN/PS) and/or PEO (1–5% w/w PEO/PS) when applicable. Membranes with no CN or PEO were used as a control. Pure water permeability of cast membranes was determined using the cross-flow cell unit. Viscosity was measured for most casting solution compositions and contact angle was measured for all membranes. The control membrane was further compared in detail to the highest permeability membranes with only CN (1.5%), only PEO (5%), 1.5% CN and 5% PEO (combination of optimal individual permeabilities), and 4.5% CN and 5% PEO (optimal combined permeability) regarding thickness, porosity, rejection, fouling resistance, surface charge, and thermal/mechanical properties. The relevance of the data presented here is to show details about methods for characterizing membranes for future comparison of performance and eventual improvement of characterization methods.

**Table:** Specifications Table

Subject area	*Environmental Engineering, Material Sciences*
More specific subject area	*Membrane Characterization, Water Treatment Technologies*
Type of data	*Tables, graphs, figures*
How data was acquired	Viscosity Measurements - Rotational Viscometer (Brookfield RVDV-E); Filtration test - Cross-Flow filtration cell (Sterlitech CF042); Contact Angle - Goniometer (Ramé-Hart Instrument Co.; Model 190 CA); Surface Roughness - Cypher Atomic Force Microscope (Asylum Research); Cross Section Images - Quanta 600FEG Environmental SEM (ESEM); Molecular Weight Cutoff - TOC analyzer (Shimadzu; TOC-VWS); Cross Flow Rejection, Surface charges and Fouling Evaluation turbidimeter (Hach; Model 2100Q), spectrophotometer (Agilent; Model 8453); Thermogravimetric analysis - TA Instruments Model TA 2950 TGA; Strain/stress analysis - Shimadzu Compact Table-top Universal Testing Machine EZTest EZ-LX; E. Coli Rejection - microscope.
Data format	*Raw and Analyzed*
Experimental factors	*Pretreatment detailed in each data subsection*
Experimental features	*Descriptions detailed in each subsection*
Data source location	*Cambridge, Massachusetts, U.S.A./São Paulo, São Paulo, Brazil*
Data accessibility	*Data is with this article*
Related research article	R. Rodrigues, J.C. Mierzwa, C. D. Vecitis, Mixed matrix polysulfone/clay nanoparticles ultrafiltration membranes for water treatment. Journal of Water Process Engineering. *V.31, 2019* [1].

**Table:** 

**Value of the Data** • Why are these data useful? Because they can be used for establishing a standard data set for membranes' characterization and performance comparison. • Who can benefit from these data? All the researchers who are involved on the field of polymeric membrane synthesis and characterization. • How can these data be used for further insights and development of experiments? Researchers can use the data for understanding the process associated with membrane production and the effect of different additives on the produced membranes.

## 1 Data

**Fig. 1:**
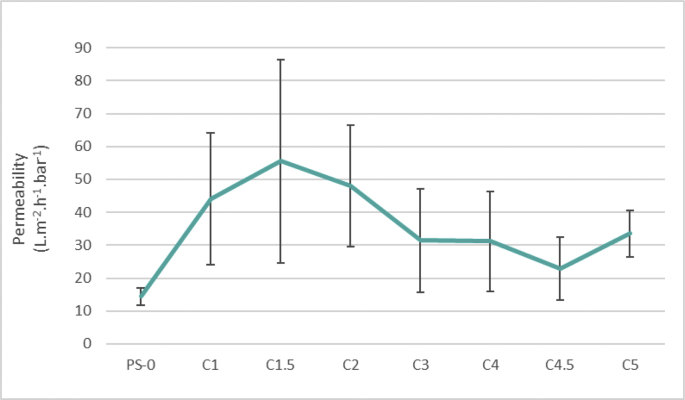
**DI permeability change with increasing clay content.** Permeability reaches a best performance and after that, decreases.

**Fig. 2:**
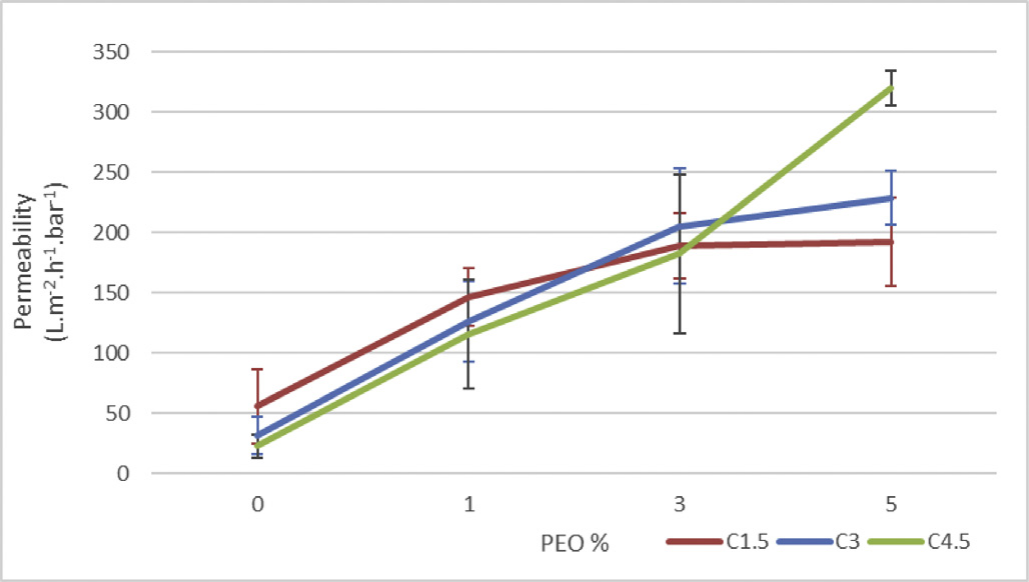
DI **permeability change with increasing PEO content.** PEO effect was more noticeable increasing permeability of 4.5% clay membranes.

**Fig. 3:**
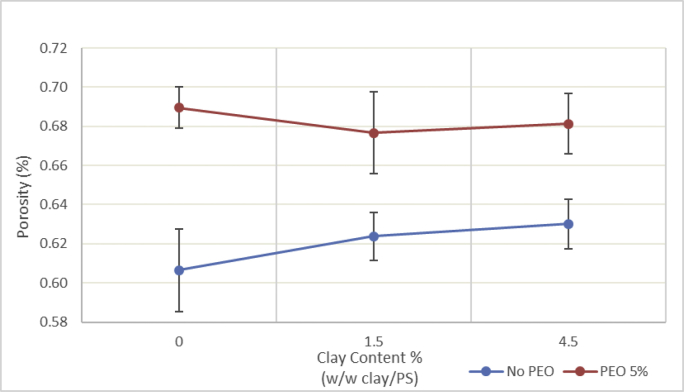
**Membrane porosity.** Membranes with PEO had a higher porosity compared to membranes with just nanoclay. nanoclay had no significant effect on membrane porosity.

**Fig. 4:**
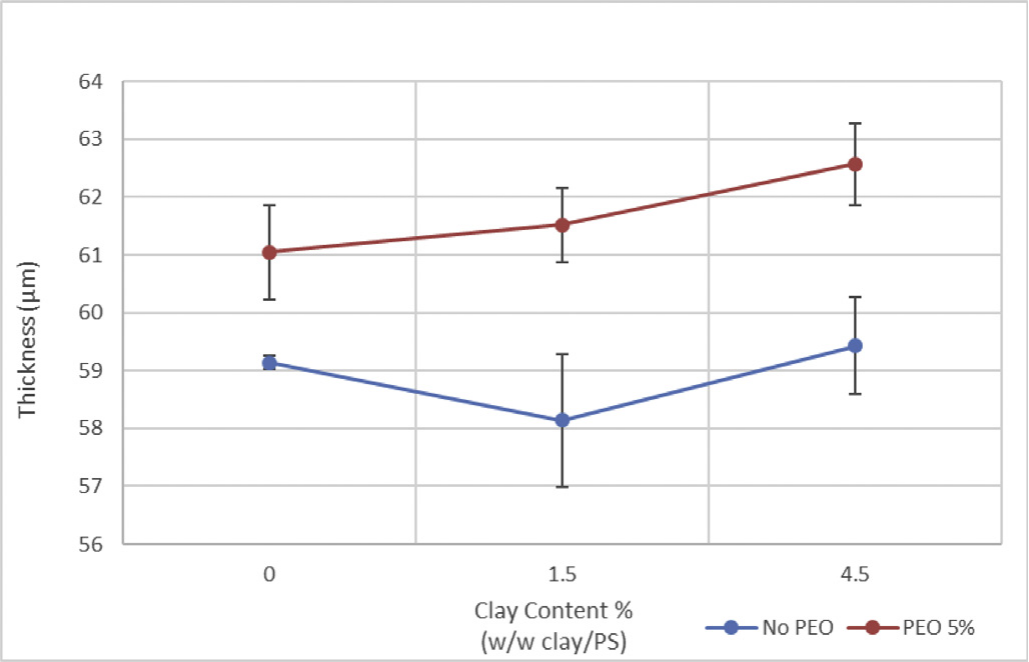
**Membrane thickness.** Membranes with PEO were thicker than non-PEO membranes. Nanoclay had no significant effect on membrane thickness.

**Fig. 5:**
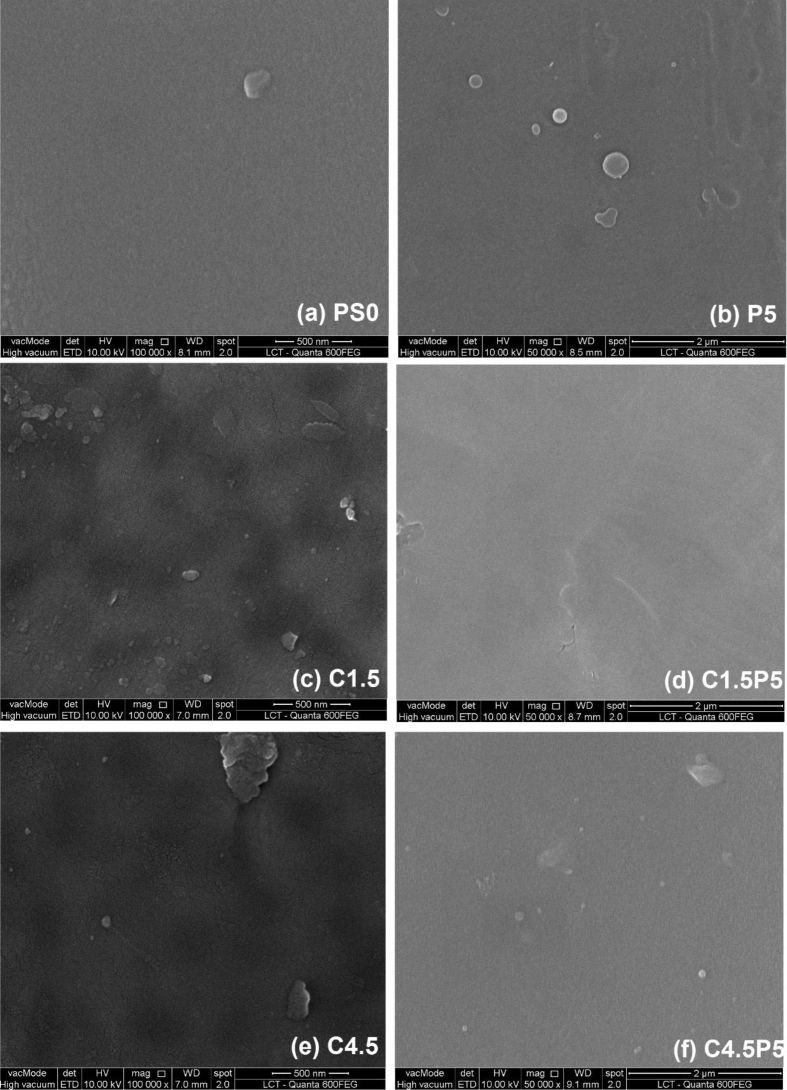
Surface SEM images of PSU, PSU/clay and PSU/clay/PEO membranes (a) Control (PS-0); (b) 5% PEO (P5); (c) 1.5% CN (C1.5); (d) 1.5% CN + 5% PEO (C1·5P5); (e) 4.5% CN (C4.5); (f) 4.5% CN + 5% PEO (C4·5P5).

**Fig. 6:**
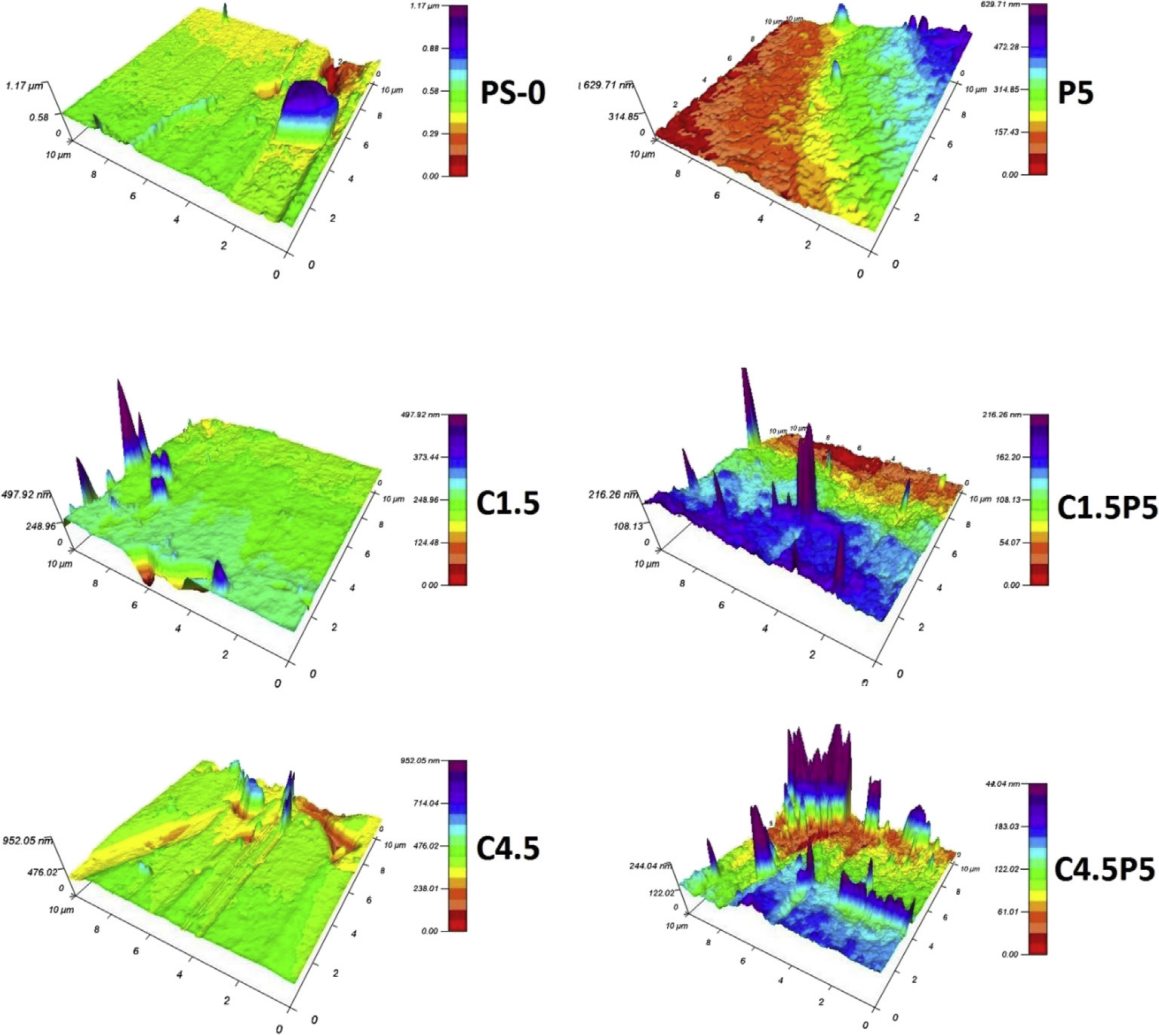
Membranes' surface roughness.

**Fig. 7:**
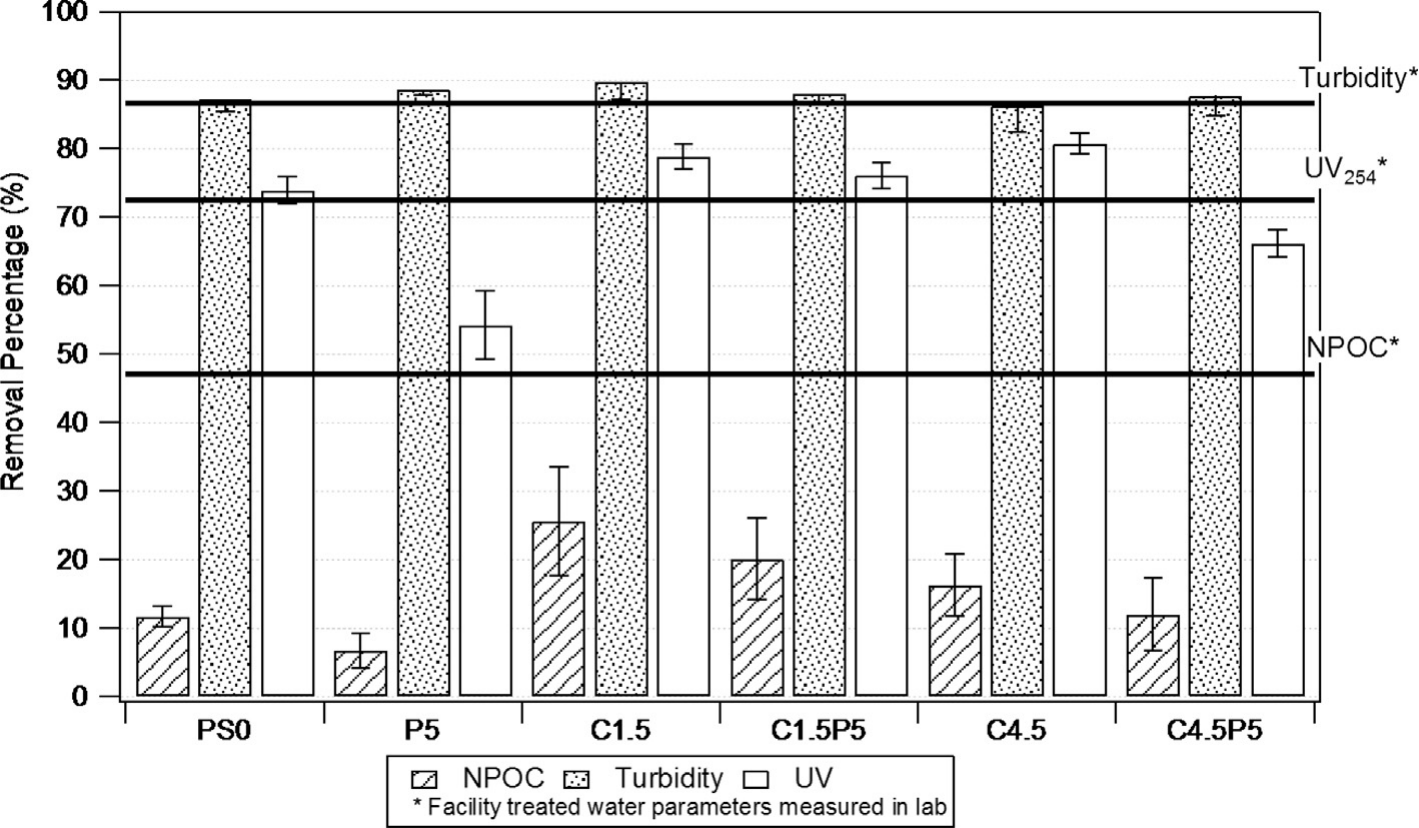
**Relative decrease in NPOC, turbidity, and UV-254 of filtered Cambridge reservoir water**. Horizontal lines refer to effluent water of the Cambridge Drinking Water Treatment Facility (measured in lab).

**Fig. 8:**
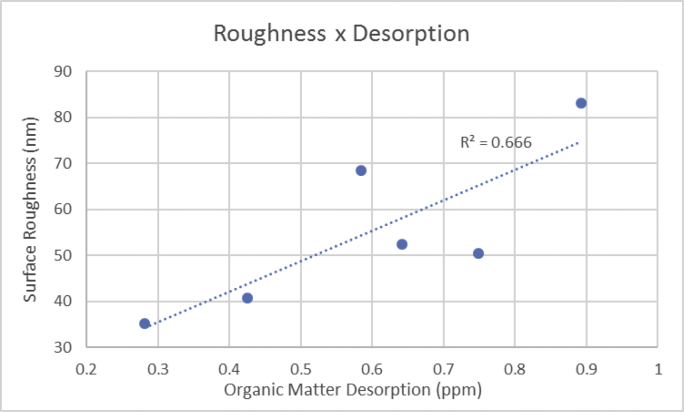
Correlation between surface roughness and organic matter desorption.

**Fig. 9:**
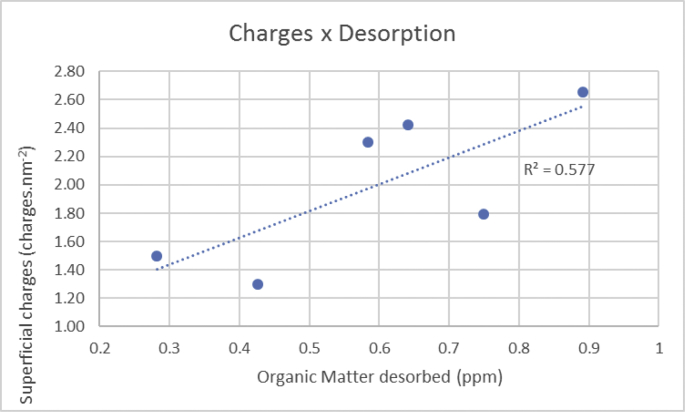
Correlation between surface charges and organic matter desorption.

**Fig. 10:**
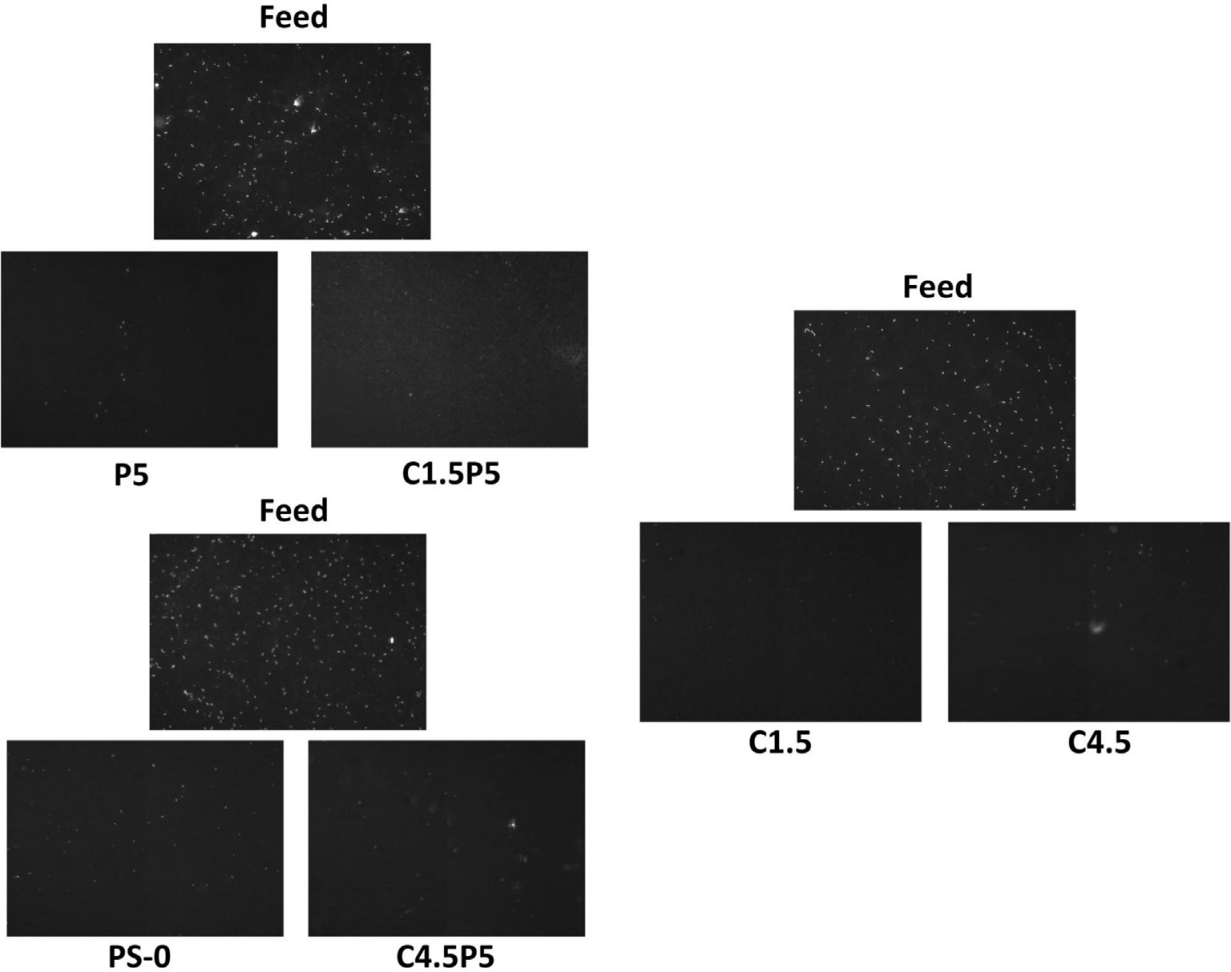
**E Coli removal**. Images showing the difference between membrane effluent and feed in terms of coliform for counting. Images have originally 278 × 200 μm and had a 40× magnification. Feed has 1 ml of E Coli concentrate solution and effluent 20 ml.

The data described include the evaluation of membrane permeability versus additive (nanoclay) content (Fig. 1, Table 1), the changes in permeability by adding a second additive (PEO) for different concentrations of first additive (Fig. 2, Table 2), membrane porosity (Fig. 3, Table 3) and thickness (Fig. 4, Table 3) for different additive contents, membrane surface images (Fig. 5) and surface roughness (Fig. 6), the results of effluent quality in terms of NPOC, Turbidity, and UV (Fig. 7, Table 4), a correlation between surface roughness (Fig. 8, Table 5) and surface charges (Fig. 9, Table 5) versus organic matter desorption, and images of E. coli in the final effluent (Fig. 10) and efficiency in E. coli removal (Table 6). Data is complemented with tables containing results of Analysis of variance for permeability (Table 7), thickness and porosity (Table 8).

**Table 1:** Permeability raw data.

	Permeability (L.m^−2^.h^−1^.bar^−1^)	Std dev
PS-0	14.38	0.026
C1	44.12	0.200
C1.5	55.52	0.309
C2	48.07	0.185
C3	31.45	0.158
C4	31.17	0.152
C4.5	22.86	0.095
C5	33.49	0.070

**Table 2:** Permeability change raw data.

	Perm. avg (L.m^−2^.h^−1^.bar^−1^)	Std dev
C1.5	0.555	0.309
C1·5P1	1.464	0.239
C1·5P3	1.887	0.272
C1·5P5	1.922	0.369
C3	0.315	0.158
C3P1	1.264	0.336
C3P3	2.050	0.479
C3P5	2.288	0.223
C4.5	0.229	0.095
C4·5P1	1.155	0.454
C4·5P3	1.822	0.658
C4·5P5	3.193	0.143

**Table 3:** Porosity and thickness raw data.

	Dry weight (g)	Wet weight (g)	Change in weight (g)	Width (cm)	Length (cm)	Area (cm2)	Thickness (μm)	Average thickness (μm)	Volume (cm3)	Porosity	Average porosity
PS0	0.366	0.126	0.24	11.7	5.6	65.52	59.14	59.19	0.388	61.93%	60.64%
	0.375	0.147	0.228	11.7	5.7	66.69	59.29		0.395	57.67%	
	0.364	0.121	0.243	11.8	5.6	66.08	59.00		0.390	62.33%	
P5	0.398	0.119	0.279	11.8	5.6	66.08	60.00	61.05	0.396	70.37%	68.96%
	0.403	0.13	0.273	11.6	5.6	64.96	62.00		0.403	67.78%	
	0.374	0.101	0.273	11.4	5.7	64.98	61.14		0.397	68.71%	
C1.5	0.358	0.135	0.223	11.3	5.6	63.28	57.71	58.14	0.365	61.06%	62.37%
	0.376	0.131	0.245	11.6	5.7	66.12	59.71		0.395	62.05%	
	0.366	0.129	0.237	11.6	5.6	64.96	57.00		0.370	64.01%	
C1·5P5	0.402	0.139	0.263	11.4	5.7	64.98	61.00	61.52	0.396	66.35%	67.67%
	0.388	0.113	0.275	11.5	5.8	66.7	62.43		0.416	66.04%	
	0.391	0.108	0.283	11.5	5.7	65.55	61.14		0.401	70.61%	
C4.5	0.374	0.146	0.228	11.3	5.6	63.28	58.29	59.43	0.369	61.82%	63.02%
	0.346	0.11	0.236	11.3	5.6	63.28	59.71		0.378	62.46%	
	0.402	0.155	0.247	11.5	5.5	63.25	60.29		0.381	64.78%	
C4·5P5	0.343	0.115	0.292	11.4	5.8	66.12	63.57	62.57	0.420	69.47%	68.13%
	0.407	0.134	0.273	11.4	5.6	63.84	62.00		0.396	68.97%	
	0.389	0.118	0.271	11.4	5.8	66.12	62.14		0.411	65.95%

**Table 4:** Raw Data for NPOC, turbidity and UV-254 in the effluent before and after treatment.

	**NPOC**							
	NPOC1	NPOC2	Average	Stdev	Rem1	Rem2	Removal	Std dev%
Raw	3.514		Treated	1.858			47.1%	
PS0	3.049	3.155	3.102	0.053	13.2%	10.2%	11.7%	1.5%
P5	3.102	3.452	3.277	0.175	11.7%	1.8%	6.7%	5.0%
C1.5	2.894	2.332	2.613	0.281	17.6%	33.6%	25.6%	8.0%
C1·5P5	3.015	2.601	2.808	0.207	14.2%	26.0%	20.1%	5.9%
C4.5	3.102	2.782	2.942	0.16	11.7%	20.8%	16.3%	4.6%
C4·5P5	2.907	3.279	3.093	0.186	17.3%	6.7%	12.0%	5.3%
	Turbidity							
	Turb.1	Turb.2	Average	Stdev	Rem1	Rem2	Removal	Std dev%
Raw	1.72		Treated	0.23			86.6%	
PS0	0.25	0.19	0.22	0.03	85.5%	89.0%	87.2%	1.7%
P5	0.18	0.21	0.195	0.015	89.5%	87.8%	88.7%	0.9%
C1.5	0.13	0.22	0.175	0.045	92.4%	87.2%	89.8%	2.6%
C1·5P5	0.18	0.23	0.205	0.025	89.5%	86.6%	88.1%	1.5%
C4.5	0.3	0.17	0.235	0.065	82.6%	90.1%	86.3%	3.8%
C4·5P5	0.26	0.16	0.21	0.05	84.9%	90.7%	87.8%	2.9%
	UV-254							
	UV-254 1	UV-254 2	Average	Stdev	Rem1	Rem2	Removal	Std dev%
Raw	0.166		Treated	0.0238			85.7%	
PS0	0.0398	0.0464	0.0431	0.0033	76.0%	72.0%	74.0%	2.0%
P5	0.0843	0.0677	0.076	0.0083	49.2%	59.2%	54.2%	5.0%
C1.5	0.038	0.0321	0.03505	0.00295	77.1%	80.7%	78.9%	1.8%
C1·5P5	0.0429	0.0366	0.03975	0.00315	74.2%	78.0%	76.1%	1.9%
C4.5	0.0344	0.0295	0.03195	0.00245	79.3%	82.2%	80.8%	1.5%
C4·5P5	0.0594	0.0528	0.0561	0.0033	64.2%	68.2%	66.2%	2.0%

**Table 5:** Organic matter desorption, surface roughness and superficial charges raw data.

Sample	Organic matter desorption (ppm)	Surface roughness (nm)	Superficial charges (charges (nm-2))
PS-0	0.650	52.4	2.41
P5	0.892	83.2	2.66
C1.5	0.272	35.3	1.53
C1·5P5	0.746	50.0	1.77
C4.5	0.416	40.7	1.29
C4·5P5	0.590	68.4	2.30

**Table 6:** *E. Coli* rejection.

Membrane	Feed (#/1 ml)	Permeate (#/20 ml)	Removal efficiency
PS0	283 ± 18	49 ± 10	99.1%
P5	295 ± 12	42 ± 10	99.3%
C1.5	224 ± 27	29 ± 7	99.4%
C1·5P5	295 ± 12	39 ± 7	99.3%
C4.5	224 ± 27	28 ± 3	99.4%
C4·5P5	283 ± 18	50 ± 11	99.1%

**Table 7:** ANOVA results for membrane permeability (5% significance level).

Compared membranes	F	F crit	*P*-value	Compared membranes	F	F crit	*P*-value
PS0–C1	9.13	4.96	1.29E-02	PS0–C3P1	44.07	5.32	1.63E-04
PS0–C1.5	7.35	4.96	2.19E-02	PS0–C3P3	63.23	5.32	4.56E-05
PS0–C2	13.02	5.32	6.90E-03	PS0–C3P5	363.74	5.32	5.92E-08
PS0–C3	5.16	4.49	3.72E-02	PS0–C4.5P1	19.74	5.32	2.16E-03
PS0–C4	10.37	5.59	1.46E-02	PS0–C4.5P3	25.98	5.32	9.33E-04
PS0–C4.5	6.45	5.59	3.86E-02	PS0–C4.5P5	1752.17	5.32	1.17E-10
PS0–C5	20.43	5.59	2.73E-03	PS0-Z1	188.79	5.32	7.59E-07
PS0–C1.5P1	123.73	5.12	1.47E-06	PS0-Z3	152.37	5.32	1.73E-06
PS0–C1.5P3	163.10	5.32	1.33E-06	PS0-Z5	211.14	5.32	4.93E-07
PS0–C1.5P5	18.40	5.12	2.02E-03

**Table 8:** ANOVA (5% significance) comparing membranes in terms of Porosity (Por) and Thickness (Thk).

Membranes compared	Variable	F	F crit	*P*-value
PS0–C1.5-C4.5	Por	1.4233	5.1432	0.3120
	Thk	1.3400	5.1432	0.3302
PS0–P5	Por	31.704	7.7086	0.0048
	Thk	10.596	7.7086	0.0312
C1.5-C1.5P5	Por	9.5951	7.7086	0.0363
	Thk	13.196	7.7086	0.0221
C4.5-C4.5P5	Por	12.988	7.7086	0.0227
	Thk	16.315	7.7086	0.0156

## 2 Experimental design, materials and methods

### 2.1 Solution viscosity

The casting solution viscosity was evaluated to assess the potential influence of additives on the phase inversion process. Viscosity is a qualitative measure of phase inversion kinetics as it is related to solvent-nonsolvent exchange rates [38,39]. Solution viscosity was measured using a medium range rotational viscometer (Brookfield RVDV-E). The casting solution was placed in a beaker and then slowly the rotational spindle is immersed to avoid bubble formation. The shear force is determined from the spindle geometry and the applied rotation rate according to equation (1):

(1) $$\eta =\frac{\tau }{\dot{\gamma }}$$

where η is the viscosity in poise, τ is the shear stress in dynes cm^−2^, and $\dot{\gamma }$ is the rate of shear (sec^−1^). The viscosity was measured at a constant shear rate of 30 RPM and temperature of 25 °C (same temperature as membrane casting). At least two different casting solution samples of each composition were evaluated.

### 2.2 Estimation of the membrane surface pore size

The identification of membrane's molecular weight cutoff (MWCO) (Mn, in Dalton) can help to understand the real pore size. Assuming constant density of the contaminant molecules, the volume of a molecule will vary linearly with the molecular weight. Depending on the shape of the molecule, the diameter can be expressed by the equation below [3,4].

(S1) $$d=\beta \;{\left(MW\right)}^{n}$$

where d (nm) is the hydrodynamic diameter of the molecule and β (nm) is the proportionality constant. n is a function of the molecular shape; and it varies from 0.33 for spheres to nearly 1.0 for linear long-chains. The specific equation for PEG is:

(S2) $$d=0.09\;{\left(MW\right)}^{0.44}$$

According to the results obtained (Fig. 7), based on 90% efficiency of removal, membranes PS0 had a MWCO between 110-120 kg mol-1, while C1.5 and C1·5P5 had MWCO of 120–130 and 190–200 kg mol-1. Therefore, PS0 has a hydrodynamic diameter between 14.9 and 15.4 nm, C1.5 between 15.4 and 16nm and C1·5P5 between 18.9 and 19.3 nm.

### 2.3 Pure water permeability – cross-flow filtration

Ultrapure water permeability was determined using the cross-flow configuration. The experiments were conducted with a bench scale system using a commercial flat-sheet cross-flow filtration cell (Sterlitech CF042) with a 34 cm^2^ filtration area. The feed solution was pumped (Micropump DJ604A) into the cross-flow filtration cell and the concentrate was recycled back to the feed reservoir. The feed solution temperature was in the range of 22–24 °C. The feed flow rate and pressure were adjusted by a valve along the concentrate pipeline and recorded by a digital flow meter (Micro-Flow FTB321D) and pressure sensor (Omega PX482A-200GI). The permeate was collected for 1 h in a graduated cylinder to determine the permeate flow rate. The remaining permeate was recycled to the feed container to maintain a constant feed solution composition. The permeate tubing was open to the atmosphere and the permeate pressure was considered to be constant at 1 bar. Permeability was calculated using the following equation:

(2) $$P=\frac{V}{A\;\times \;\Delta t\;\times \;\Delta P}$$

where P is the permeability (L m^−2^ h^−1^ bar^−1^), V is the volume of permeate collected (L), A is the effective membrane area (m^2^), Δt is the sampling time (h), and ΔP is the transmembrane pressure (bar).

### 2.4 Contact angle measurements

Contact angle measurements were determined with a goniometer (Ramé-Hart Instrument Co.; Model 190 CA) using the sessile drop technique. The membrane preparation and contact angle measurement were carried out according to the ISO-15989 standard procedure [40]. Three samples of each membrane were evaluated and at least 30 measurements were made on each sample.

### 2.5 Surface roughness

Surface roughness measurements were conducted using a Cypher Atomic Force Microscope (Asylum Research) operating in amplitude modulation with a silicon tip (Bruker OTESPA) at a resonance frequency of 340 kHz and nominal tip radius of 7 nm. Images of 10 × 10 μm were acquired using 1 V amplitude and a 0.6 V set point amplitude at a scan rate of 1 Hz. The image refinement to create 3D surfaces was made using the software Argyle Light and the data extracted was analyzed with the R13.17.101 extension for IgorPro®. The images were flattened with order 1 (to minimize errors by curvature and slope) and the root mean squared (RMS) roughness was calculated using the deviation of data.

### 2.6 Morphology analysis

Membrane cross-sections images were obtained using a Quanta 600FEG Environmental Scanning Electron Microscope (ESEM) operated in secondary electron detection mode with a 10 kV accelerating voltage. All membrane samples were coated with a modular high vacuum coating system (BAL-TEC MED 020) resulting in an ∼10 nm platinum layer after 120–160 s of deposition at 43 mA current. To image cross-sections, the membrane samples were first immersed in liquid nitrogen for 30 s and then cleanly snapped.

Membrane porosity was determined following previously reported wet/dry weight methods. The wet weight was measured after removing the superficial water with two polyester/cellulose wipers (VWR International) and the dry weight was measured after drying the samples. The porosity was calculated using the following equation:

(3) $$\varepsilon =\frac{\frac{m_{1}-m_{2}}{\rho_{w}}}{V_{m}}$$

where m_1_ and m_2_ (g) are the wet and dry weights, ρ_w_ (g cm^−3^) is the density of water, V_m_ (cm^3^) is the membrane volume, and ε (%) is the bulk porosity. The volume, V_m_, was calculated by multiplying the sample area by its thickness, which was measured by a digital micrometer (Fowler Tools and Instruments; 1.27 to 25,400 μm).

### 2.7 Membrane molecular weight cut-off

To determine the molecular weight cut-off (MWCO), membranes were challenged in the same cross-flow cell used for ultrapure water permeability using PEG feed solutions of molecular weight 10, 50, 90, and 203 kg mol^−1^. The feed and permeate PEG concentrations were determined using the non-purgeable organic carbon (NPOC) method on a TOC analyzer (Shimadzu; TOC-VWS). The feed contained approximately 20 mgC L^−1^ of the chosen PEG (NPOC equivalent) in ultrapure water. Samples were collected after 1 h of operation for at least three membrane samples. Rejection (R) was defined by the following equation:

(4) $$R\left(\text{\%}\right)=\left(1-\frac{C_{p}}{C_{f}}\right)\times 100$$

where C_p_ and C_f_ are the NPOC concentrations in the permeate and feed, respectively.

### 2.8 Cross flow rejection

A similar procedure for MWCO evaluation was used to determine sodium alginate (10 mgC L^−1^) rejection with effluent measurements made after 2 hours of continuous operation.

To evaluate natural surface water treatment efficacy, the influent was used from Fresh Pond, a local drinking water reservoir, and permeate measurements were made after 2 hours of operation. NPOC, turbidity, and UV_254_ of the feed and permeate were measured to evaluate the treatment efficiency. The turbidity was measured using a portable turbidimeter (Hach; Model 2100Q). The UV_254_ absorption was measured using a UV–Visible spectrophotometer (Agilent; Model 8453).

### 2.9 Fouling evaluation

For evaluation of natural surface water fouling potential, the membranes were challenged for 8 hours at 1 bar transmembrane pressure and the permeability decrease was compared to initial ultrapure water experiments. After fouling, the membranes were chemically cleaned using an alkaline (pH 11) feed solution for 30 minutes and subsequently rinsed with copious amounts of ultrapure water. After cleaning, the membranes were challenged again with ultrapure water to evaluate the flux recovery (as an estimative of irreversible fouling). At least three membrane samples were evaluated.

In order to evaluate the amount of organic matter adsorbed by the membrane surface, membrane samples were challenged with natural surface waters using the same conditions as the fouling potential measurement. After running for 8 hours, 9 cm^2^ squares were cut, immersed in solutions at pH 2, 7, and 11 and kept in a rotating shaker (New Brunswick Scientific, E24) at 60 rpm for 6 hours. The NPOC of the solution containing these squares was measured to quantify the desorbed organic matter.

### 2.10 Negative surface charge evaluation

The membrane negative surface charge was quantified following a previously reported procedure [2]. Briefly, 4 × 4 cm membrane samples were taped to a glass slide such that 9 cm^2^ of the top surface of the membrane was exposed. The membrane surface was then coated with a thin film of 0.5 mM toluidine blue and 15 mM NaCl solution at pH 6–7 for ∼60 s. The dye solution was washed off with copious amounts of 15 mM NaCl and then the membrane samples were placed in a 15 mM NaCl water bath for 4 h to remove any weakly attached dye. The 9 cm^2^ samples were then cut from the larger coupons and placed in a 20-mL glass vial containing 10 mL of 0.2 M NaCl at pH 2 (HCl). The vial was stirred in a rotating shaker at 50 rpm for 30 min at 35 °C. The low pH protonates any negatively-charged surface groups and releases the electrostatically bound positively-charged dye, which was then quantified by UV–vis spectroscopy (*λ*_max_ = 634 nm; *ε* = 45,200 cm^−1^ M^−1^). The negative surface charge density (n^−^) in number per nm^2^ was quantified using equation (5):

(5) $$n^{-}=\frac{A\;\times \;V\;\times \;N}{\varepsilon \;\times \;SA}$$

where A is the absorption at 634 nm, V = 0.01 L is the volume of extraction solution, N = 6.022 × 10^23^ is Avogadro's number, and SA = 9 × 10^14^ nm^2^ is the surface area of the measured membrane sample. At least four samples of a selected membrane were evaluated for negative surface charge.

### 2.11 Thermal and mechanical properties

Thermogravimetric analysis (TGA) was completed using a TA Instruments Model TA 2950 TGA under nitrogen and oxygen flows at 10 mL min^−1^ each. Samples were placed on a platinum pan and the temperature was increased at 10 °C min^−1^ from 25 to 750 °C.

Strain/stress analysis was completed on a Shimadzu Compact Table-top Universal Testing Machine EZTest EZ-LX at 25 °C with a crosshead speed of 5 mm min^−1^. Samples had a cross section of thickness x 5 × 15 mm. At least 3 samples were analyzed for average tensile strength, Young's modulus, and elongation at break.

### 2.12 E. Coli rejection

*E. Coli* (w3110) was used to measure bacterial removal efficiency. Bacteria were harvested at mid-exponential phase and then centrifuged and resuspended in 0.9% NaCl saline solution twice prior to addition to the feed solution. The bacteria concentration in the feed and permeate was determined using fluorescence microscopy. Briefly, the solution (1 mL for the feed and 20 mL for the permeate) was vacuum filtered onto a polycarbonate membrane (Sterlitech PCTE), and the bacteria were stained with 4’,6-diamidino-2-phenylindole (DAPI) for 5 minutes and then analyzed (excitation/emission of 358/461 nm). The prepared filter was transferred to the fluorescence microscope and imaged at 40× magnification. At least five random points on the filter were imaged (278 × 200 μm) and analyzed for cell enumeration.
